# Novice Physician Ultrasound Evaluation of Pediatric Tricuspid Regurgitant Jet Velocity

**DOI:** 10.5811/westjem.2020.3.45882

**Published:** 2020-06-24

**Authors:** Zachary W. Binder, Sharon E. O’Brien, Tehnaz P. Boyle, Howard J. Cabral, Sepehr Sekhavat, Joseph R. Pare

**Affiliations:** * Boston Medical Center, Boston University School of Medicine, Department of Pediatrics, Boston, Massachusetts; † Boston University School of Public Health, Department of Biostatistics, Boston, Massachusetts; ‡ Boston Medical Center, Boston University School of Medicine, Department of Emergency Medicine, Boston, Massachusetts

## Abstract

**Introduction:**

Pulmonary hypertension, associated with high mortality in pediatric patients, is traditionally screened for by trained professionals by measuring a tricuspid regurgitant jet velocity (TRJV). Our objective was to test the feasibility of novice physician sonographers (NPS) to perform echocardiograms of adequate quality to exclude pathology (defined as TRJV > 2.5 meters per second).

**Methods:**

We conducted a cross-sectional study of NPS to assess TRJV by echocardiogram in an urban pediatric emergency department. NPS completed an educational course consisting of a didactic curriculum and hands-on workshop. NPS enrolled a convenience sample of patients aged 7–21 years. Our primary outcome was the proportion of echocardiograms with images of adequate quality to exclude pathology. Our secondary outcome was NPS performance on four image elements. We present descriptive statistics, binomial proportions, kappa coefficients, and logistic regression analysis.

**Results:**

Eight NPS completed 80 echocardiograms. We found 82.5% (95% confidence interval [CI], 74.2–90.8) of echocardiograms had images of adequate quality to exclude pathology. Among image elements, NPS obtained a satisfactory, apical 4-chamber view in 85% (95% CI, 77.1–92.9); positioned the color box accurately 65% (95% CI, 54.5–75.5); optimized TRJV color signal 78.7% (95% CI, 69.8–87.7); and optimized continuous-wave Doppler in 55% (95% CI, 44.1–66.0) of echocardiograms.

**Conclusion:**

NPS obtained images of adequate quality to exclude pathology in a majority of studies; however, optimized acquisition of specific image elements varied. This work establishes the basis for future study of NPS assessment of TRJV pathology when elevated pulmonary pressures are of clinical concern.

## INTRODUCTION

### Background

Pulmonary hypertension, while rare in children and young adults, carries a 25% five-year mortality.^1^ Pulmonary hypertension is a clinical consideration during various pediatric emergency department (PED) presentations including syncope and pulmonary embolism.^2^,^3^ Thirty-six percent of children with idiopathic or familial pulmonary hypertension first present with syncope.^1^ The gold standard for diagnosing pulmonary hypertension is cardiac catheterization.^4^ Alternatively, cardiologists estimate pulmonary artery pressure by measuring a tricuspid regurgitant jet velocity (TRJV) during transthoracic echocardiography. An elevated TRJV of > 2.5 meters per second (m/s) is a surrogate measure of elevated pulmonary artery pressure *in lieu* of invasive cardiac catheterization (TRJV pathology).^5^,^6^ While a pathologic TRJV is rare in children and young adults, approximately 68–86% of healthy individuals will have a non-pathologic TRJV (detectable jet velocity up to 2.5 m/s).^7^–^9^ The remaining 14–32% of healthy children and young adults will have no tricuspid regurgitant jet.

To date, TRJV has only been reliably measured by cardiologists and ultrasound technicians with cardiologist oversight. Unfortunately, this level of expertise, which is the current standard of care, is often unavailable to emergency physicians at times when patients may have concerning clinical presentations.^10^ Emergency physician sonographers have previously been shown to accurately assess and measure many components of a point-of-care echocardiogram.^11^–^16^ Point-of-care ultrasound, in comparison to comprehensive ultrasound, is an abbreviated examination performed by a medical professional at the bedside, which is used to identify either the presence or absence of specific pathologic findings. Previous studies have used a combination of didactic instruction and practical training to teach specific components of a point-of-care echocardiogram to emergency physicians.^11^–^16^ To our knowledge, the ability of novice physician sonographers (NPS) to perform assessments of TRJV has not been previously studied. As a result, we performed this pilot study aimed at determining feasibility.

### Goals of Investigation

Our study objectives were to 1) test the feasibility of NPS to perform echocardiograms in the PED of adequate quality to exclude pathology (defined as TRJV > 2.5 m/s) as determined by a blinded pediatric cardiologist, and 2) identify patient and NPS characteristics associated with adequately performed echocardiograms.

## METHODS

### Study Design

We conducted a prospective cross-sectional study from April 2018 to October 2018 in an urban, tertiary care facility with an annual census of 24,000 PED visits. The facility is a Level I trauma center with an accredited pediatric emergency medicine (PEM) fellowship and EM residency. The facility operates a separate PED that cares for patients through 21 years of age.

### Patient Population

NPS screened and enrolled a convenience sample of patients aged 7–21 years who presented to the PED. Medical records were reviewed prior to approaching subjects for enrollment. We excluded patients who were critically ill, non-English speaking, or were younger than seven years of age. Patients less than seven years of age were excluded at the recommendation of the study’s primary cardiologist to limit exclusions based on patient intolerance. All other patients were eligible for enrollment. The patient or patient’s guardian (for minors under 18 years of age) provided informed consent prior to enrollment. All patients less than 18 years of age provided verbal assent. The Boston University Medical Campus and Boston Medical Center Institutional Review Board approved this study.

**Population Health Research Capsule**

What do we already know about this issue?

*Emergency physician sonographers have previously been shown to accurately assess many components of a point-of-care echocardiogram*.

What was the research question?

Are novice physician sonographers able to perform echos that exclude tricuspid regurgitant jet (TRJ) pathology

What was the major finding of the study?

*Pediatric cardiologist rated 82.5% of echos performed to be of adequate quality to exclude TRJ pathology*.

How does this improve population health?

*This pilot study establishes the basis for future investigation of novice assessment of TRJ pathology when elevated pulmonary pressures are of clinical concern*.

### Novice Physician Sonographer Population

We aimed to analyze the performance of NPS, which we defined as individuals who had performed fewer than 50 lifetime echocardiograms. This number was selected based on the 2016 ACEP policy statement.^17^ The primary investigator (PI) recruited EM residents, pediatric EM fellows, and pediatric EM attendings as unpaid volunteers to participate as NPS based on their limited echocardiography experience. All of the institution’s first-year EM residents (14), eligible pediatric EM fellows (2), and eligible pediatric EM attendings (5) were first contacted by email, followed by in-person recruitment if interest in participation was expressed.

### Educational Intervention

**Figure 1:**
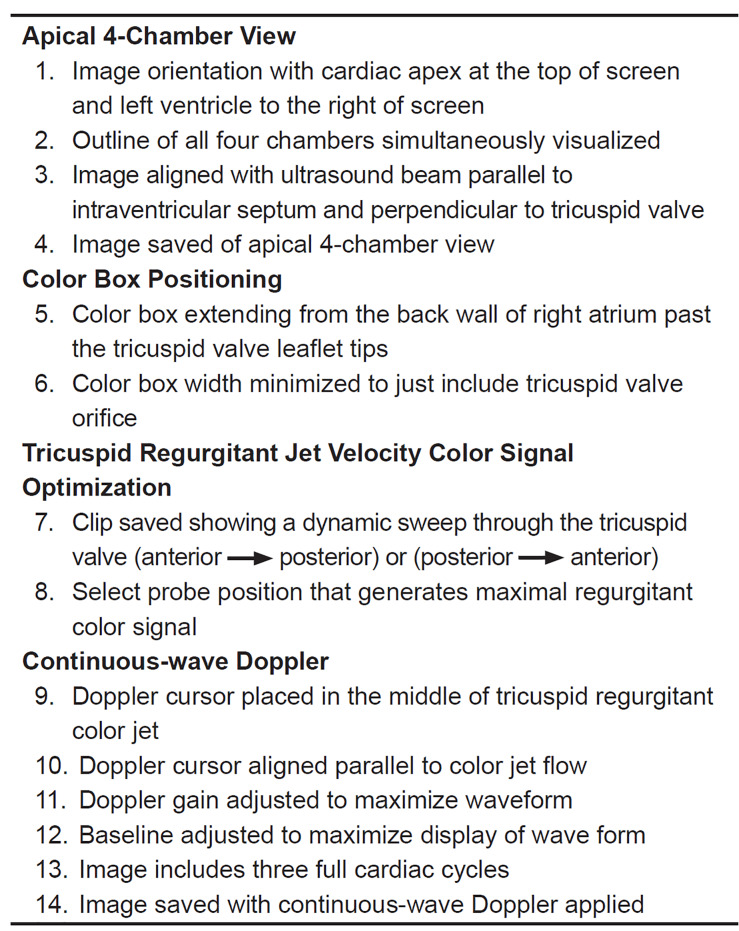
Tricuspid regurgitant jet velocity curriculum.

**Figure 2:**
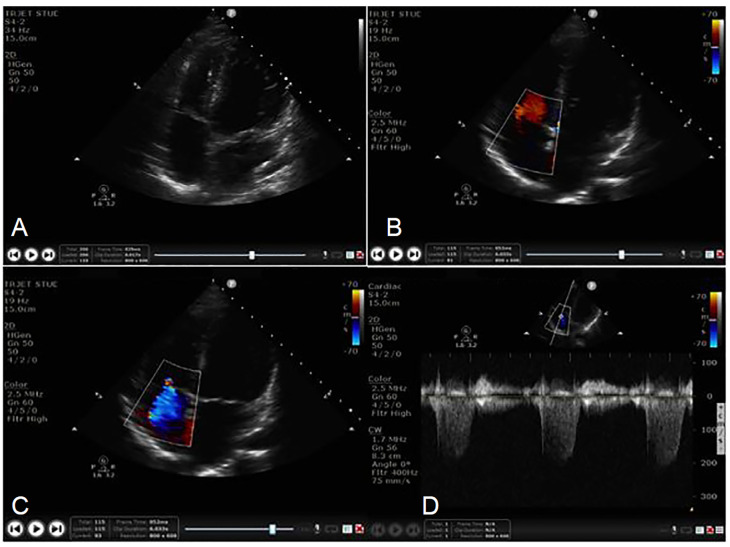
Tricuspid regurgitant jet velocity image elements. 2A. Apical 4-Chamber, 2B. Color box positioning, 2C. Tricuspid regurgitant jet signal optimization, 2D. Continuous-wave Doppler interrogation.

Prior to participation in the study, NPS completed a three-hour educational course combining a didactic curriculum (30 minutes) and hands-on workshop (2.5 hours). The educational course taught NPS the steps required to obtain TRJV images. The didactic curriculum used still-image and video-clip modalities to demonstrate a stepwise approach to obtaining a TRJV. As no curriculum for TRJV image acquisition exists, the study’s principal investigator (PI) and primary pediatric cardiologist (SO) created a curriculum through a comprehensive multistep process. A literature review of pediatric echocardiography reference materials led to the generation of an initial curriculum.^18^–^20^ A panel of three independent pediatric cardiologists from outside the study institution reviewed the curriculum and provided feedback. These cardiologists had no role in curriculum generation, or image rating. We obtained consensus on all proposed modifications using modified Delphi methodology.^21^ The final curriculum contained four elements: 1) apical 4-chamber view; 2) color box positioning; 3) TRJV color signal optimization; and 4) continuous-wave Doppler interrogation (Figure 1 and Figure 2). The study’s PI and primary pediatric cardiologist (SO) administered the didactic curriculum.

Immediately following the didactic curriculum, NPS participated in a hands-on workshop consisting of deliberate practice with direct feedback from experts (professional cardiac sonographer and certified pediatric cardiologist). All NPS were required to complete a proctored echocardiogram during the workshop where an expert assessed performance. The proctored examination required the successful completion of all four image elements. Proctored echocardiograms established the novice’s ability to obtain TRJV images prior to his or her participation in the study.

### Image Rating

The study’s primary cardiologist, SO, blinded to NPS and patient identity, reviewed each recorded study. The cardiologist first reviewed all images to determine whether they could confidently exclude pathology based on the images provide. The cardiologist then performed a secondary analysis where individual image elements were assessed (Figure 1). The cardiologist recorded these results on an electronic score sheet. To assess inter-rater reliability a second pediatric cardiologist, not otherwise involved in the study, independently reviewed 20% of all study echocardiograms.

### Sample Size Estimation

We predicted 80% of echocardiograms would have images of adequate quality to exclude pathology (TRJV > 2.5 m/s). Based on this estimate, we required 80 echocardiograms to generate a 95% confidence interval (CI) with a lower bound percentage of 72.6%. Therefore, each of the eight NPS was asked to perform a minimum of 10 echocardiograms over a six-month period.

### Data Collection

NPS recorded still images and video clips in a protocolized fashion using a Philips SPARQ (Philips Healthcare, Bothell, WA) ultrasound machine. Images automatically transferred wirelessly to Qpath (Telexy Healthcare, Maple Ridge, BC) software, a program for storage and management of ultrasound examinations. NPS obtained all images and clips using a standardized imaging preset with a phased array probe (S4-2). We de-identified all study images at the time of acquisition with a study identification.

NPS completed a standardized data collection form after each echocardiogram. The study’s PI abstracted patient information including gender, age, ethnicity, vital signs, and body mass index (BMI) from the electronic health record. The study’s PI transcribed clinical data into Research Electronic Data Capture (REDCap) (Nashville, TN).^22^

### Statistical Analysis

Descriptive statistics, binomial proportions, and kappa coefficients were performed to analyze the data. We tested for patient and NPS characteristics associated with adequately performed echocardiograms using logistic regression analysis. All statistical analyses were performed using SAS 9.4 software (SAS Institute, Cary, NC).

## RESULTS

### Demographics

NPS consented 75 eligible patients for participation during the six-month study period. Two consented patients did not have echocardiograms performed because of time constraints. Ultimately 73 patients, ages 7–21 years, provided 80 echocardiograms for analysis (seven patients provided two echocardiograms by different NPS). Table 1 details patient and NPS characteristics.

### Primary Outcome

**Figure 3:**
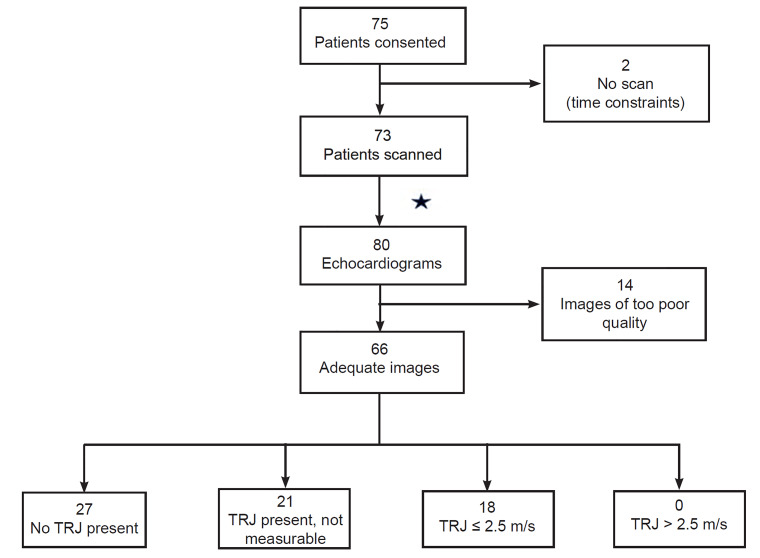
Flowchart demonstrating patient enrollment and image rating as determined by pediatric cardiologist. ★Seven patients were scanned by two sonographers. *TRJ*, tricuspid regurgitant jet; *m/s*, meters per second.

The study’s primary pediatric cardiologist rated 66 of 80 (82.5%, 95% CI, 74.2–90.8) echocardiograms to have images of adequate quality to exclude pathology. The remaining 14 of 80 were deemed to be of too poor quality to assess for the presence of TRJV. Of 66 echocardiograms, 27 (40.9%) had no TRJ present, 21 (31.8%) had a present but not measurable TRJ, and 18 (27.3%) had a measurable TRJV less than or equal to 2.5 m/s. None of the echocardiograms had a TRJV greater than 2.5 m/s (Figure 3).

### Secondary Outcomes

Of the four image elements, the proportion of satisfactorily completed elements ranged from 55% (95% CI, 44.1–66.0) for the interrogation of continuous-wave Doppler to 85% (95% CI, 77.1–92.9) for the acquisition of apical 4-chamber view (Table 2A). To complete the examinations, NPS took an average of 2.5 minutes (interquartile range [IQR] 1.6 – 4.7) from time of first to last saved ultrasound image timestamp. NPS performed an average of 11 echocardiograms (IQR 8–13) (Table 2B).

For the variables selected to test association with image quality, younger patient age was associated with improved echocardiogram adequacy (odds ratio [OR] 0.64; 95% CI, 0.41–0.99; p=0.04). No association was found between patient gender or NPS level of clinical training and adequacy of images (Table 2C). There was fair agreement (κ = 0.25) between the two pediatric cardiologists when assessing the primary outcome, the ability to exclude pathology.

## DISCUSSION

The study found 82.5% of echocardiograms to have images of adequate quality to exclude pathology, TRJV >2.5 m/s. We believe this provides preliminary evidence NPS can perform adequate TRJV studies following a brief educational intervention. This is in line with prior studies showing novices can accurately assess and measure other focused components of a point-of-care echocardiogram.^11^–^16^

It is important to note for this study it was possible to obtain images of adequate quality to exclude pathology without performing each image element optimally. For example, NPS may have failed to adjust the display to produce a textbook image; however, the images may still have been adequate to exclude pathology when reviewed by the cardiologist. The objective of our secondary analysis was to determine which image elements were more difficult for NPS. They were most successful in acquiring an apical 4-chamber view (85%), and least successful in optimally interrogating continuous-wave Doppler (55%). The interrogation of continuous-wave Doppler is an advanced skill and this study likely represented the NPS’s first exposure to this function. These results provide further support that ultrasound performance is dependent on repeated exposure and practice.^17^,^23^,^24^

We found a statistically significant association between younger patient age and echocardiograms with images of adequate quality. This association may be explained by thinner chest walls in younger patients; however, the study was inadequately powered to evaluate this relationship. Furthermore, the study was inadequately powered to determine a relationship between patient BMI and image adequacy.

We found a fair level of inter-rater agreement. Liem et al demonstrated an inter-rater kappa of 0.45 (moderate agreement) when measuring TRJV.^25^ Their study analyzed measurements obtained by expert sonographers that were then interpreted by cardiologists. It is possible that images obtained by novices have a greater range of quality leading to a lower inter-rater agreement. Both our study and that by Liem et al suggest expert agreement regarding the assessment of TRJV is varied.

It is notable that 82.5% of echocardiograms in our study had images of adequate quality to exclude pathology. As an initial pilot study, the first step was to determine ability regarding image acquisition. We recognize that future study of NPS interpretation and incorporation of their echocardiograms into clinical care is needed.

## LIMITATIONS

As a convenience sample of non-critically ill patients from a single center, this may limit the study’s generalizability. Our findings will require external validation in a future larger study prior to clinical implementation. While no patients in the study had a TRJV greater than 2.5 m/s, this was not unexpected. NPS successfully detected TRJVs at lower velocities. Further study would be required to demonstrate a NPS’s ability to rule in pathology. We feel that our patient population is representative of a general population because prior studies of healthy children and young adults found a similar distribution of TRJV findings.^7^–^9^

The primary study outcome, adequacy of images, is subjective and therefore subject to bias. We found no objective assessment tool available for the purposes of our study. In an attempt to limit bias, we blinded the raters to patient and NPS information. In prior studies on this subject, experts have only been able to generate moderate inter-rater agreement suggesting the lack of an ideal assessment mechanism.

The study’s primary cardiologist (SO) helped develop the initial curriculum and served as one of the study’s image raters. Three independent cardiologists not involved in the study, via a modified Delphi approach, finalized this curriculum. It is possible that by having the primary cardiologist assist with the initial curriculum development and grading the images bias was introduced into this study. We believe this would be limited by having the external pediatric cardiologists provide consensus on approving the final curriculum. Additionally, the two cardiologists were blinded to both patient and NPS identity when rating images.

The study’s protocol did not incorporate pulsed-wave Doppler prior to continuous-wave Doppler analysis. This is an accepted practice in echocardiography and has been used in prior studies on the topic.^26^ The risk in excluding pulsed-wave Doppler is overestimating the TRJV through contamination of signal from extremely rare intracardiac shunting lesions. Identification of shunting lesions, while outside the scope of this study, represents pathology that one would ideally not miss. We do not believe the decision had an impact on our study’s results.

## CONCLUSION

There is currently no way to easily assess pulmonary hypertension in the PED setting. The ability to assess for pulmonary hypertension in the PED could assist in the management of multiple patient presentations including syncope and pulmonary embolism. While this study was performed in a PED, we believe the results and potential clinical implications would also apply to an adult population. It is important to note that in the era of the electronic health record, if an emergency physician could perform a study and review the images with a cardiologist it might improve the quality of subspecialty input and referrals at the time of ED presentations. We acknowledge these evaluations are best done in consultation with cardiology colleagues and not as a replacement for their expertise. This study suggests that NPS can obtain images of adequate quality to evaluate TRJV in the absence of pathology (TRJV > 2.5 m/s) after a brief educational intervention. This work establishes the basis for future study of novice assessment of TRJV pathology when elevated pulmonary pressures are of clinical concern.

## Figures and Tables

**Table 1:** Characteristics of patients and novice physician sonographers (NPS) in study to determine whether NPS could obtain point-of-care echocardiogram images of adequate quality to exclude pathology.

Patient characteristics (n = 73)	Median [IQR]
Age (years)	19 [17–20]
BMI (kg/m^2^)*	23.9 [21.9–27.5]
Gender (female)	40/73 (54.8%; 95% CI, 43.3–66.3)
Ethnicity (Hispanic)	19/73 (26.0%; 95% CI, 15.9–36.1)

Patient vital signs (n = 73)	Median [IQR]

Temperature (°F)	99.0 [97.3–98.7]
Heart rate (beats/minute)	79 [68–88]
Respiratory rate (breaths/minute)	18 [16–18]
Systolic blood pressure (mmHg)	120 [112–124]
Diastolic blood pressure (mmHg)	74 [68–78]
Oxygen saturations (%)	99 [98–100]

Novice physician sonographer level of training (n = 8)	n (%)

Emergency medicine residents	2 (25.0%)
Pediatric emergency medicine fellows	2 (25.0%)
Pediatric emergency medicine attendings	4 (50.0%)

* N = 52 for this variable.

*IQR*, interquartile range; *kg/m^2^*, kilograms per meter squared; °*F*, degrees Fahrenheit; *mmHg*, millimeters of mercury; *BMI*, body mass index; *CI*, confidence interval.

**Table 2:** Echocardiography results.

2A. Rating of TRJV image elements	Ratio of satisfactory completion	95% CI
Apical 4-chamber	68/80 (85.0%)	(77.1–92.9)
Color box positioning	52/80 (65.0%)	(54.5–75.5)
TRJ signal optimization	63/80 (78.7%)	(69.8–87.7)
Continuous-wave doppler	44/80 (55.0%)	(44.1–66.0)

2B. Echocardiogram characteristics	Median	[IQR]

Scans per sonographer	11	[8–13]
Time to complete ultrasound study (minutes)	2.5	[1.6–4.7]

2C. Association with adequate echocardiogram*	OR [95% CI]	P-value

Patient age	0.64 [0.41–0.99]	0.04
Patient gender	0.58 [0.16–2.13]	0.41
Sonographer: resident vs attending	0.75 [0.15–3.78]	0.72
Sonographer: fellow vs attending	0.84 [0.17–4.18]	0.83

* Logistic regression analysis.

*CI*, confidence interval; *TRJV*, tricuspid regurgitant jet velocity; *IQR*, interquartile range; *OR*, odds ratio.
